# The mitochondrial protein TIMM44 is required for angiogenesis in vitro and in vivo

**DOI:** 10.1038/s41419-023-05826-9

**Published:** 2023-05-05

**Authors:** Zhou-rui Ma, Hong-Peng Li, Shi-zhong Cai, Sheng-Yang Du, Xia Chen, Jin Yao, Xu Cao, Yun-Fang Zhen, Qian Wang

**Affiliations:** 1 Department of Burns and Plastic Surgery, Children’s hospital of Soochow University, Suzhou, China; 2 Suzhou Key Laboratory of Children’s Structural Deformities, Suzhou, China; 3 Kunshan Hospital of Chinese Medicine, Affiliated Hospital of Yangzhou University, Kunshan, China; 4 Department of Child and Adolescent Healthcare, Children’s Hospital of Soochow University, Suzhou, China; 5 Department of Orthopedics, Xuzhou First People’s Hospital, Xuzhou, China; 6 Department of Anesthesiology, Children’s hospital of Soochow University, Suzhou, China; 7 The Affiliated Eye Hospital, Nanjing Medical University, Nanjing, China; 8 Department of Urology, Children’s Hospital of Soochow University, Suzhou, China; 9 Department of Orthopedics, Children’s hospital of Soochow University, Suzhou, China

**Keywords:** Stress signalling, Mechanisms of disease

## Abstract

The mitochondrial integrity and function in endothelial cells are essential for angiogenesis. TIMM44 (translocase of inner mitochondrial membrane 44) is essential for integrity and function of mitochondria. Here we explored the potential function and the possible mechanisms of TIMM44 in angiogenesis. In HUVECs, human retinal microvascular endothelial cells and hCMEC/D3 brain endothelial cells, silence of TIMM44 by targeted shRNA largely inhibited cell proliferation, migration and in vitro capillary tube formation. TIMM44 silencing disrupted mitochondrial functions in endothelial cells, causing mitochondrial protein input arrest, ATP reduction, ROS production, and mitochondrial depolarization, and leading to apoptosis activation. TIMM44 knockout, by Cas9-sgRNA strategy, also disrupted mitochondrial functions and inhibited endothelial cell proliferation, migration and in vitro capillary tube formation. Moreover, treatment with MB-10 (“MitoBloCK-10”), a TIMM44 blocker, similarly induced mitochondrial dysfunction and suppressed angiogenic activity in endothelial cells. Contrarily, ectopic overexpression of TIMM44 increased ATP contents and augmented endothelial cell proliferation, migration and in vitro capillary tube formation. In adult mouse retinas, endothelial knockdown of TIMM44, by intravitreous injection of endothelial specific TIMM44 shRNA adenovirus, inhibited retinal angiogenesis, causing vascular leakage, acellular capillary growth, and retinal ganglion cells degeneration. Significant oxidative stress was detected in TIMM44-silenced retinal tissues. Moreover, intravitreous injection of MB-10 similarly induced oxidative injury and inhibited retinal angiogenesis in vivo. Together, the mitochondrial protein TIMM44 is important for angiogenesis in vitro and in vivo, representing as a novel and promising therapeutic target of diseases with abnormal angiogenesis.

## Introduction

Vascular dysfunction is a common pathogenesis mechanism of a number of diseases, such as ischemic stroke, heart failure, diabetes, cancer, and retinal disorders [1, 2]. Oxygen and nutrient shortage following vascular disorder shall cause imbalance of metabolic demand and supply [3, 4], which could initiate angiogenesis process [3, 4]. VEGF (vascular endothelial growth factor) and other growth factors, as well as various cytokines and other factors could also activate endothelial cells and promote angiogenesis [3–7].

Angiogenesis is a dynamic and energy-consuming process [3–7]. To form new blood vessels, endothelial cells in the lumen of blood vessels shall change from the static state to proliferative state [7–9]. Mitochondria are endothelial signal centers, which regulate endothelial functions and promote angiogenesis by coordinating energy metabolism, reactive active oxygen (ROS), calcium concentration, and apoptosis [10–12].

In addition to providing energy for cells, mitochondria are vital in regulating key cell processes, including cell differentiation, signaling transduction, and apoptosis, as well as cell growth and cell cycle progression [13–18]. The functional state of mitochondria is vital for maintaining mitochondrial membrane potential, mitochondrial membrane channel integrity, Ca^2+^ concentration, respiratory chain complex activity, and ATP synthesis. Mitochondrial dysfunction is mainly manifested in changes in mitochondrial morphology and structure, decreased ATP production, excessive production of ROS and oxidative injury, mtDNA damage and apoptosis activation [16–18]. Thus, endothelial mitochondria are key signaling hubs regulating angiogenesis other key endothelial cell functions [11, 19].

Translocase of inner mitochondrial membrane (TIMM) 44, or TIMM44, is a mitochondrial protein locating at the inner mitochondrial membrane [20, 21]. TIMM44 anchors the mitochondrial heat shock protein 70 (mtHsp70) protein to TIMM23 protein complex. This process is ATP-dependent [22–24]. TIMM44 modulates mitochondrial pre-proteins’ import to the mitochondrial matrix [22, 23]. This process requires appropriate inner membrane potential and ATP production through mtHsp70 [22, 23]. TIMM44-overexpressed transgenic mice were protected from Type-II diabetes [20]. Mitochondrial genes, including *mitofusin-1 (Mfn1), mitofusin-2 (Mfn2)*, and *optic atrophy 1 (Opa1)*, were upregulated in TIMM44 transgenic mice [20, 25]. TIMM44 overexpression inhibited ROS production and oxidative injury, whereas increasing ATP production [25, 26]. Here we explored the potential function and the possible mechanisms of TIMM44 in angiogenesis in vitro and in vivo.

## Materials and methods

### Reagents, chemicals, and antibodies

Polybrene, puromycin, medium, and serum were obtained from Sigma-Aldrich (St. Louis, MO). Antibodies for Opa1(#80471), mitofusin-1 (Mfn1, #14739), and mitofusin-2 (Mfn2, #11925) were purchased from Cell Signaling Tech (Danvers, MA). Other antibodies were reported previously [25]. All fluorescence probes were from Thermo-Fisher Invitrogen (Shanghai, China). MB-10 (“MitoBloCK-10”) was reported previously [25].

### Cell culture and transfection

Human umbilical vein endothelial cells (HUVECs), human retinal microvascular endothelial cells (hRMECs) and hCMEC/D3 brain endothelial cells were reported in the previous studies [27–30]. Cells were cultured in described medium and were maintained under the pro-angiogenic state [29, 30].

### Gene silencing or overexpression

The lentiviral particles with the TIMM44 shRNA (shTIMM44-seq1/2, two different sequences), the scramble control shRNA, the TIMM44-overexpressing construct, or the empty vector (“Vec”) were described early [25]. The viral particles, at MOI = 15, were added directly to the endothelial cells maintained under the polybrene-containing medium. After 48 h, puromycin-containing medium was added to select stable cells for another 96 h. TIMM44 silencing or overexpression was verified at mRNA and protein levels. Expression of Opa1, Mfn1, or Mfn2 in HUVECs was through the same procedure.

### CRISPR/Cas9-induced TIMM44 knockout

Endothelial cells were seeded at 65–70% confluence and were transduced with a Cas9-expressing construct (Genechem, Shanghai, China). The lentiviral particles with the CRISPR/Cas-9 TIMM44 knockout (KO) construct, encoding sgRNA sequence against human TIMM44 (as reported previously [25]), were added directly to Cas9-expressing endothelial cells. After 48 h, puromycin-containing medium was added to select stable cells for additional 96 h. TIMM44 KO in the single stable cells was verified at mRNA and protein levels.

### Mitochondrial protein import assay

The detailed protocols were described elsewhere [25, 31]. In brief, the [^35^S]-methionine plus cysteine-labeled Su9-dihydrofolate reductase (DHFR) fusion protein was utilized [25]. Mitochondria (200 µg/mL) were dissolved to the import buffer [31] and import reaction was started by adding the fusion protein in the mitochondria mix. After 15 min, the import of the fusion protein was terminated. Mitochondria were pelleted, dissolved and input proteins measured via SDS-PAGE and autoradiography [25].

### Thiobarbituric acid reactive substance assaying of lipid peroxidation

A commercial thiobarbituric acid reactive substance (TBAR) kit (Cayman Chemical, MI) was utilized to test lipid peroxidation in total cellular lysates (25 μg per sample). The kit specifically quantified lipid peroxidation intensity by colorimetrically measuring malondialdehyde (MDA). The TBAR intensity optical density (OD) was tested at 545 nm with the reference of 600 nm. It was expressed in nmol per mg of total protein, with its value normalized to that of control.

### Other cellular functional studies

Nuclear EdU (5-ethynyl-2′-deoxyuridine)/DAPI (4,6-diamidine-2′-phenylindole dihydrochloride) staining (testing cell proliferation), in vitro cell migration “Transwell” assay, in vitro cell invasion “Matrigel Transwell” assay, and in vitro capillary tube formation as well as nuclear TUNEL (terminal deoxynucleotidyl transferase dUTP nick end labeling)/DAPI staining of cell apoptosis, the Caspase-3 activity assay, JC-1 (tetrachloro-tetraethylbenzimidazolylcarbocyanine iodide) staining of mitochondrial potential and ROS detection by CellROX and DCF-DA (dichlorofluorescein diacetate) staining were described in detail in previous studies [25, 29, 30, 32, 33]. The quantitative real-time PCR (qRT-PCR) and Western blotting were described early [28, 29, 33–36]. Isolation of mitochondrial fraction lysates and mitochondrial-null lysates was through the described protocol [25, 37]. The cellular ATP contents were examined as described [38]. The mitochondrial complex I activity was tested via a commercial kit (Abcam) [25]. The superoxide dismutase (SOD) activity in fresh mouse retinal tissues was measured by a SOD ELISA kit (Thermo-Fisher Invitrogen, Shanghai, China) using the attached protocols. The uncropped blotting images were shown in Fig. S1.

### Animal studies

The adult C57BL/6 mice (hale male half female, 4–5 week old, 18.3–19.2 g) were provided by the SLAC Laboratory Animal Center (Shanghai, China). Mice were maintained under standard conditions and were anesthetized as described [29, 30]. Approximately 0.1 μL AAV or MB-10 (or vehicle control) was intravitreously injected into the vitreous cavity with the needle directly above the optic nerve head [29, 30]. NeuN (Neuronal Nuclei) immunofluorescence staining of retinal ganglion cells (RGCs) in the mouse retinal slides, retinal vasculature detection by isolectin B4 (IB4) staining, retinal trypsin digestion assaying of acellular capillaries, Evans blue (EB) staining of vascular leakage were described in detail in previous studies [29, 30]. The Institutional Animal Care and Use Committee and the Ethic Committee of Soochow University approved the protocols, and animal studies were in according to the ARVO (Association for Research in Vision and Ophthalmology) statement.

### Statistical analyses

Data, expressed as means ± standard deviation (SD), were all normally distributed. One-way ANOVA plus a Scheffe’s f-test (for comparison of three or more groups, SPSS 23.0), or the two-tailed unpaired *t* test (for comparison of two groups, Excel 2007) were utilized to examine significance. *P* values < 0.05 were considered statistically significant.

## Results

### TIMM44 silencing inhibits proliferation, migration, and tube formation in cultured endothelial cells

**Fig. 1:**
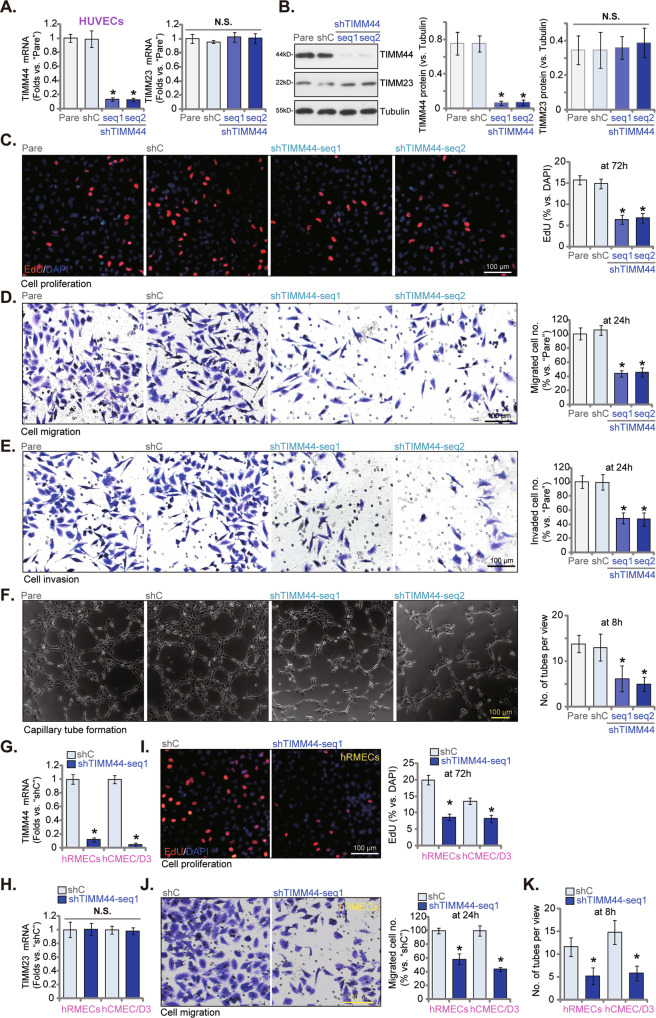
TIMM44 silencing inhibits proliferation, migration and tube formation in cultured endothelial cells. HUVECs (**A**–**F**), the human retinal microvascular endothelial cells (hRMECs, **G**–**K**), or hCMEC/D3 brain endothelial cells (**G**–**K**), with the applied shRNA (shTIMM44-seq1, shTIMM44-seq2 or shC), were cultured, and expression of *TIMM23*/*TIMM44* mRNA and proteins tested (**A**, **B**, **G**, and **H**); The endothelial cells were further cultivated for indicated time periods, cell proliferation, in vitro cell migration and invasion were examined by the nuclear EdU staining (**C**, **I**), “Transwell” (**D**, **J**) and “Matrigel Transwell” (**E**) assays, respectively; the in vitro capillary tube formation was tested and formed tubes quantified (**F**, **K**). “Pare” stands for the parental control cells. Data were presented as mean ± standard deviation (SD, *n* = 5). *P < 0.05 versus “Pare”/“shC” cells. “N. S.” stands for non-statistical differences (*P* > 0.05). The experiments were repeated five times with similar results obtained. Scale bar = 100 μm.

First lentivirus encoding two shRNAs against human *TIMM44*, shTIMM44-seq1 and shTIMM44-seq2 (with different sequences [25]), were individually transfected to cultured HUVECs, and following selection by puromycin single stable HUVECs formed. *TIMM44* mRNA expression decreased over 80% in shTIMM44-expressing HUVECs (Fig. 1A), where *TIMM23* mRNA levels were unchanged (Fig. 1A). TIMM44 protein downregulation was detected as well in HUVECs with TIMM44 shRNA (Fig. 1B), and TIMM23 protein expression again unchanged (Fig. 1B). shTIMM44 HUVECs and HUVECs with scramble non-sense control shRNAs (“shC”) were maintained at the pro-angiogenic state under the describe medium [29]. As shown shRNA-induced silencing of TIMM44 inhibited HUVEC proliferation and substantially decreased the percentage of EdU positively-stained nuclei (Fig. 1C). Moreover, in vitro cell migration and invasion, tested separately by “Transwell” (Fig. 1D) and “Matrigel Transwell” (Fig. 1E) assays, were largely inhibited after TIMM44 silencing (Fig. 1D, E). Furthermore, the in vitro capillary tube formation ability was robustly decreased in shTIMM44-expressing HUVECs (Fig. 1F).

To both human retinal microvascular endothelial cells (hRMECs) and hCMEC/D3 brain endothelial cells [29], shTIMM44-seq1-expressing lentivirus were added. Stable cells were formed with puromycin selection. *TIMM44* mRNA silencing was detected in shTIMM44-seq1-expresssing endothelial cells (Fig. 1G), and *TIMM23* mRNA levels unchanged (Fig. 1H). The nuclear EdU staining assay and “Transwell” assay results revealed that shRNA-induced knockdown of TIMM44 robustly inhibited cell proliferation (Fig. 1I) and migration (Fig. 1J) in the endothelial cells. The in vitro capillary tube formation was also largely inhibited after TIMM44 silencing in hRMECs and hCMEC/D3 cells (Fig. 1K). These results supported that TIMM44 silencing inhibited in vitro angiogenesis in cultured endothelial cells.

### TIMM44 silencing disrupts mitochondrial functions in endothelial cells

**Fig. 2:**
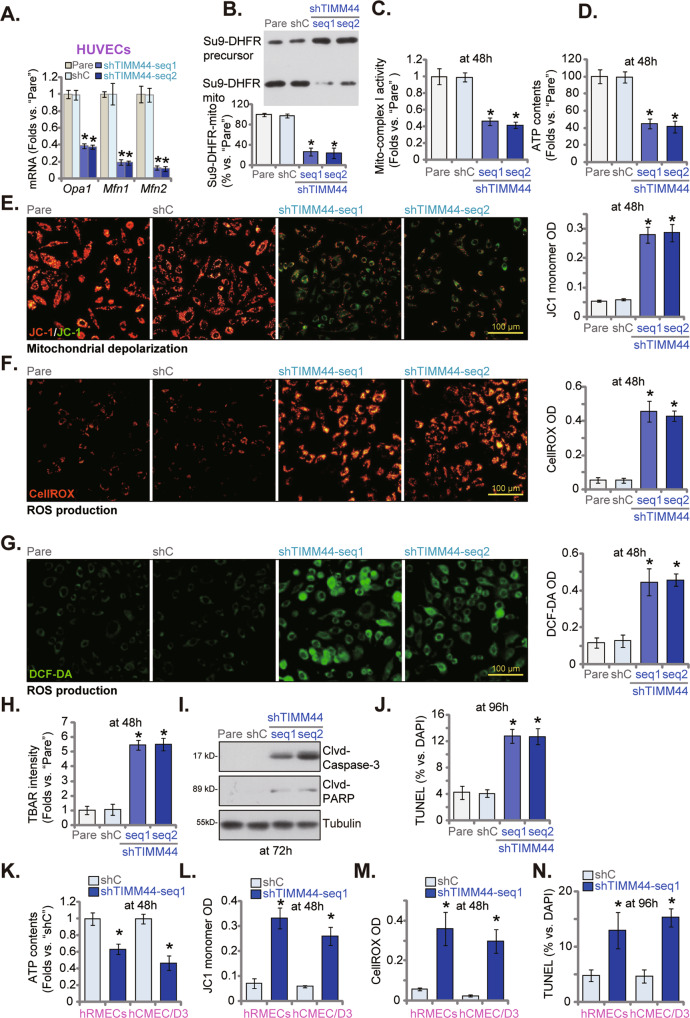
TIMM44 silencing disrupts mitochondrial functions in endothelial cells. HUVECs (**A**–**J**), the human retinal microvascular endothelial cells (hRMECs, **K**–**N**), or hCMEC/D3 brain endothelial cells (**K**–**N**), with the applied shRNA (shTIMM44-seq1, shTIMM44-seq2 or shC), were cultured (**A**); The isolated mitochondria (200 µg/mL) were incubated with [^35^S]-methionine and cysteine-labeled precursor Su9-DHFR protein, and mitochondria protein import stopped by adding trypsin after 15 min. The precursor Su9-DHFR protein (“Su9-DHFR-precursor”) and mitochondrial Su9-DHFR protein (“Su9-DHFR-mito”) were examined through SDS-PAGE (**B**), with the relative Su9-DHFR-mito intensity quantified (**B**); The endothelial cells were further cultivated for indicated time periods, the mitochondrial complex I activity (**C**) and ATP contents (**D**, **K**) were examined; The mitochondrial membrane potential reduction (JC-1 staining, **E**, **L**), ROS (DCF-DA/CellROX intensity, **F**, **G**, **M**) and lipid peroxidation (TBAR activity, **H**) were examined by the mentioned assays. Cell apoptosis was tested via measuring Clvd-caspase-3/Clvd-PARP levels (**I**) and TUNEL-positive nuclei percentage (**J**, **N**). Data were presented as mean ± standard deviation (SD, *n* = 5). *P < 0.05 versus “Pare”/“shC” cells. “N. S.” stands for non-statistical differences (*P* > 0.05). The experiments were repeated five times with similar results obtained. Scale bar = 100 μm.

TIMM44 regulates import of mitochondrial pre-proteins to the mitochondrial matrix [22, 23] and is important for maintaining the integrity and functions of mitochondria, we examined whether TIMM44 silencing altered mitochondrial functions in endothelial cells. In cultured HUVECs, viral shRNA-induced knockdown of TIMM44 (by shTIMM44-seq1 and shTIMM44-seq2, see Fig. 1) substantially reduced mRNA expression of TIMM44-dependent mitochondrial genes, including *Opa1*, *Mfn1* and *Mfn2* [20, 25] (Fig. 2A). TIMM44 silencing remarkably suppressed Su9-DHFR fusion protein’s import to the mitochondria (Fig. 2B), and 70–80% mitochondrial protein import reduction was observed after TIMM44 knockdown in HUVECs (Fig. 2B). Moreover, the mitochondrial complex I activity was decreased after TIMM44 silencing (Fig. 2C). Cellular ATP contents were decreased as well in shTIMM44 HUVECs (Fig. 2D).

TIMM44 shRNA induced mitochondrial depolarization and resulted in JC-1 green monomers accumulation in HUVECs (Fig. 2E). Enhanced ROS production and oxidative injury were observed in TIMM44-silenced HUVECs (Fig. 2F, G). The CellROX red fluorescence intensity (Fig. 2F) and the DCF-DA green fluorescence intensity (Fig. 2G) were both substantially increased in shTIMM44 HUVECs. Moreover, TBAR activity assay results suggested enhanced lipid peroxidation in HUVECs with TIMM44 silencing (Fig. 2H).

Further studies revealed that TIMM44 silencing induced moderate but significant apoptosis in HUVECs. Compared to the shC HUVECs, levels of cleaved (Clvd-) caspase-3 and Clvd-poly (ADP-ribose) polymerase (PARP) were increased in HUVECs with shTIMM44-seq1 or shTIMM44-seq2 (Fig. 2I). In addition, the TUNEL positively-stained nuclei ratio was increased in TIMM44-silenced HUVECs, implying apoptosis activation (Fig. 2J). In both hRMECs and hCMEC/D3 cells, TIMM44 silencing by viral shTIMM44-seq1 (see Fig. 1) similarly resulted in ATP depletion (Fig. 2K) and mitochondrial depolarization (increased JC-1 green monomer intensity, Fig. 2L). Supporting ROS production, the CellROX fluorescence intensity was increased in TIMM44-silenced endothelial cells (Fig. 2M). Moderate apoptosis activation, evidenced by increased nuclear TUNEL staining, was observed in the endothelial cells (Fig. 2N). These results clearly supported that TIMM44 silencing disrupted mitochondrial functions in cultured endothelial cells.

### TIMM44 KO inhibits pro-angiogenic activity in HUVECs

**Fig. 3:**
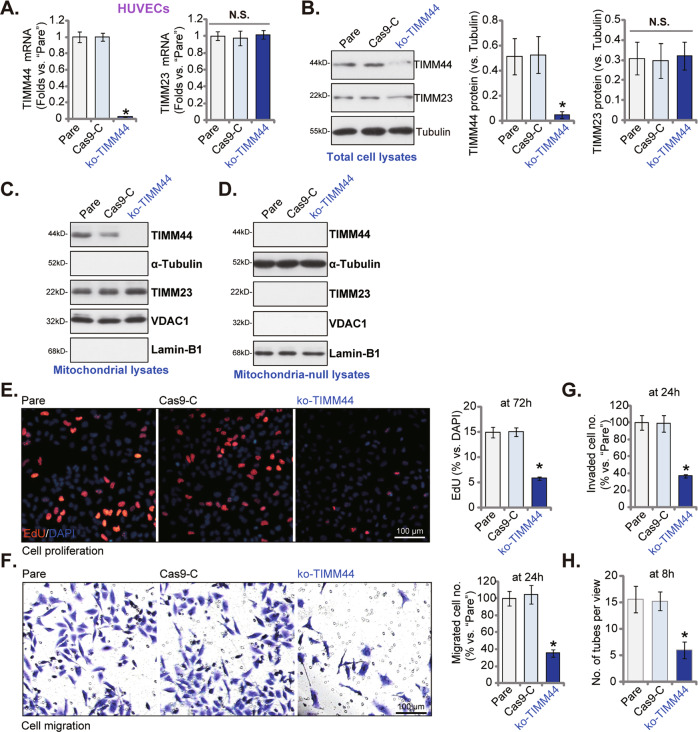
TIMM44 KO inhibits pro-angiogenic activity in HUVECs. HUVECs, expressing the Cas9 construct plus the CRISPR/Cas9-TIMM44-KO construct (“ko-TIMM44”) or the CRISPR/Cas9 empty vector (“Cas9-C”), were cultured, expression *TIMM23*/*TIMM44* mRNA and listed proteins (in both total cellular lysates, mitochondrial lysates, and mitochondrial-null lysates) was shown (**A**–**D**). HUVECs were further cultivated for indicated time periods, cell proliferation, in vitro cell migration, and invasion were examined by the nuclear EdU staining (**E**), “Transwell” (**F**), and “Matrigel Transwell” (**G**) assays, respectively, with results quantified; The in vitro capillary tube formation was tested and formed tubes were quantified (**H**). Data were presented as mean ± standard deviation (SD, *n* = 5). *P < 0.05 versus “Pare” cells. “N. S.” stands for non-statistical differences (*P* > 0.05). The experiments were repeated five times with similar results obtained. Scale bar = 100 μm.

To further support the role of TIMM44 in endothelial cells, the CRISPR/Cas9 gene-editing method was utilized to knockout TIMM44. Briefly, a lentiviral CRISPR/Cas9-TIMM44-KO construct was stably transduced to Cas9-expressing HUVECs [29], and single stable cells formed by puromycin selection and TIMM44 KO verification. These cells were named as ko-TIMM44 HUVECs, and *TIMM44* mRNA (Fig. 3A) and protein (Fig. 3B) expression depleted. *TIMM23* mRNA and protein expression, as expected, was unchanged in ko-TIMM44 HUVECs (Fig. 3A, B). The mitochondrial fractions of the HUVECs were isolated and tested. TIMM44 protein was only enriched in the mitochondrial lysates (Fig. 3C), indicated by the presence of the mitochondrial protein VDAC1 (voltage-dependent anion-selective channel 1) (Fig. 3C), but the absence of the nuclear marker protein Lamin-B1 and the cytosol marker protein α-Tubulin (Fig. 3C), Once again, the mitochondrial TIMM44, but not TIMM23, was depleted in ko-TIMM44 HUVECs (Fig. 3C). TIMM44, TIMM23, and VDAC1 were not detected in the mitochondria-null lysates of HUVECs, whereas Lamin-B1 and α-Tubulin were both present (Fig. 3D).

In cultured HUVECs, CRISPR/Cas9-induced TIMM44 KO remarkably attenuated in vitro cell proliferation, migration and invasion, tested separately by nuclear EdU staining (Fig. 3E), “Transwell” (Fig. 3F) and “Matrigel Transwell” (Fig. 3G) assays, respectively. Moreover, TIMM44 KO largely inhibited in vitro capillary tube formation in HUVECs (see quantified results in Fig. 3H). The lentiviral CRISPR/Cas9-KO control construct, Cas9-C, failed to alter TIMM44/TIMM23 expression (Fig. 3A–D) and HUVEC functions (Fig. 3E–H).

### TIMM44 KO disrupts mitochondrial functions in HUVECs

**Fig. 4:**
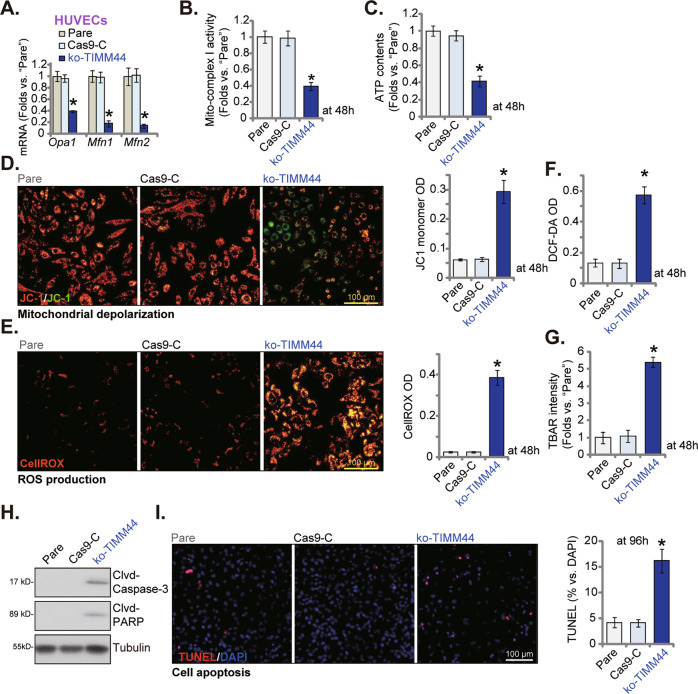
TIMM44 KO disrupts mitochondrial functions in HUVECs. HUVECs, expressing the Cas9 construct plus the CRISPR/Cas9-TIMM44-KO construct (“ko-TIMM44”) or the CRISPR/Cas9 empty vector (“Cas9-C”), were cultured, expression of listed mRNAs was tested (**A**); The mitochondrial complex I activity (**B**) and ATP contents (**C**) were examined; The mitochondrial membrane potential reduction (JC-1 staining, **D**), ROS intensity (DCF-DA/CellROX intensity, **E** and **F**) and lipid peroxidation (TBAR intensity, **G**) were examined by the mentioned assays. Cell apoptosis was tested via measuring Clvd-caspase-3/Clvd-PARP levels (**H**) and TUNEL-positive nuclei (**I**). Data were presented as mean ± standard deviation (SD, n = 5). *P < 0.05 versus “Pare” cells. “N. S.” stands for non-statistical differences (*P* > 0.05). The experiments were repeated five times with similar results obtained. Scale bar = 100 μm.

We proposed that TIMM44 KO should disrupt mitochondrial functions in endothelial cells. TIMM44-dependent mitochondrial genes, *Opa1*, *Mfn1*, and *Mfn2*, were significantly decreased in ko-TIMM44 HUVECs (Fig. 4A). Moreover, the mitochondrial complex I activity (Fig. 4B) and the ATP contents (Fig. 4C) were decreased following TIMM44 KO in HUVECs. Further studies showed that JC-1 green monomers were significantly increased in ko-TIMM44 HUVECs, indicating mitochondrial depolarization (Fig. 4D). Moreover, ROS contents were increased in TIMM44 KO HUVECs, as the CellROX red fluorescence intensity (Fig. 4E) and DCF-DA green fluorescence intensity (Fig. 4F) were both increased. Increased TBAR intensity implied lipid peroxidation in ko-TIMM44 HUVECs (Fig. 4G). Supporting apoptosis activation, we found that levels of Clvd-caspase-3/Clvd-PARP (Fig. 4H) and the TUNEL-positive nuclei percentage (Fig. 4I) were augmented in TIMM44 KO HUVECs. The Cas9-C control construct failed to disrupt mitochondrial functions (Fig. 4A–G) nor inducing apoptosis (Fig. 4H, I) in HUVECs. Thus, TIMM44 KO disrupted mitochondrial functions and provoked apoptosis in HUVECs.

We also examined whether re-expression of Mfn1, Mfn2, and Opa1 could rescue TIMM44-depleted endothelial cells. Therefore, to the ko-TIMM44 HUVECs, the Opa1-expressing lentiviral construct, the Mfn1-expressing lentiviral construct and the Mfn2-expressing lentiviral construct were consecutively transduced. Stable cells were then formed after selection by puromycin (“ko-TIMM44 + Opa1/Mfn1/Mfn2 Vec”). Mfn1, Mfn2 and Opa1 protein expression was restored in these cells (Fig. S2A). Importantly, re-expression of Mfn1, Mfn2, and Opa1 in HUVECs attenuated TIMM44 depletion-induced ATP reduction (Fig. S2B) and mitochondrial depolarization (Fig. S2C). Moreover, cell proliferation (Fig. S2D), migration (Fig. S2E), invasion (Fig. S2F), and capillary tube formation (Fig. S2G) were partially restored in the “ko-TIMM44 + Opa1/Mfn1/Mfn2 Vec” HUVECs. These results supported that downregulation of Mfn1, Mfn2, and Opa1 should be one important mechanism of TIMM44 depletion-induced angiogenesis inhibition in HUVECs.

### The TIMM44 blocker MB-10 inhibits endothelial cell angiogenesis in vitro

**Fig. 5:**
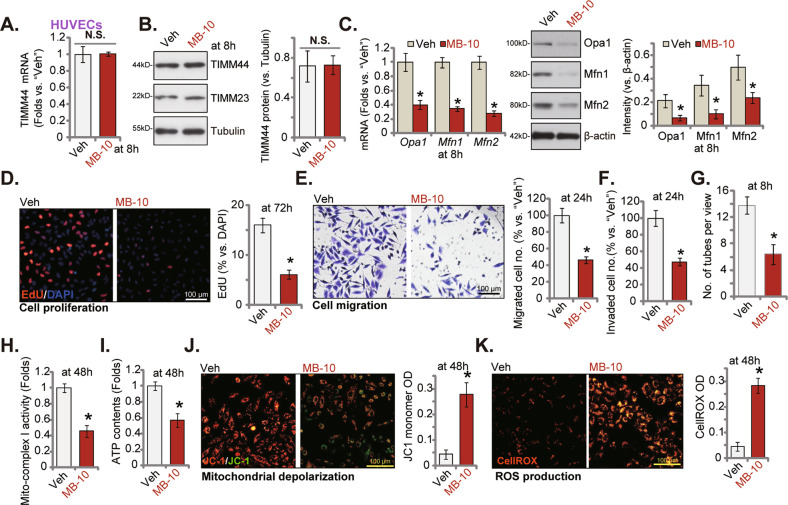
The TIMM44 blocker MB-10 inhibits endothelial cell angiogenesis in vitro. HUVECs were treated with MitoBloCK-10 (MB-10, for 25 μM) or the vehicle control (“Veh”, 0.1% DMSO), and cells were further cultivated for designated time, expression of listed mRNAs and proteins (**A**–**C**) was tested. Cell proliferation, in vitro cell migration and invasion were examined by the nuclear EdU staining (**D**), “Transwell” (**E**), and “Matrigel Transwell” (**F**) assays, respectively; The in vitro capillary tube formation was tested and formed tubes quantified (**G**); The mitochondrial complex I activity (**H**) and ATP contents (**I**) were examined; The mitochondrial membrane potential reduction (JC-1 green monomers intensity, **J**) and ROS intensity (CellROX intensity, **K**) were examined as well. Data were presented as mean ± standard deviation (SD, *n* = 5). *P < 0.05 versus “Veh” cells. “N. S.” stands for non-statistical differences (*P* > 0.05). The experiments were repeated five times with similar results obtained. Scale bar = 100 μm.

MB-10 (“MitoBloCK-10”) binds to a specific pocket in the C-terminal domain of TIMM44, suppressing mitochondrial protein import and disrupting mitochondrial functions [31]. We therefore examined the potential role MB-10 in HUVECs. Treatment with MB-10 failed to alter *TIMM44* mRNA (Fig. 5A) and protein (Fig. 5B) expression in HUVECs. *TIMM23* mRNA and protein levels were unchanged in MB-10-treated HUVECs (Fig. 5A, B). Yet mRNA and protein expression levels of TIMM44-dependent mitochondrial genes, *Opa1*, *Mfn1,* and *Mfn2*, were downregulated in MB-10-treated HUVECs (Fig. 5C). MB-10 inhibited cell proliferation and reduced EdU-positive nuclei percentage in HUVECs (Fig. 5D). Mover, HUVEC in vitro cell migration (Fig. 5E) and invasion (Fig. 5F) were largely suppressed following treatment of the TIMM44 blocker. The number of formed capillary tubes was also decreased after treatment with MB-10 (Fig. 5G). Analyzing mitochondrial functions, we showed that the TIMM44 blocker reduced the mitochondrial complex I activity (Fig. 5H) and decreased ATP contents (Fig. 5I) in HUVECs. MB-10 resulted mitochondrial depolarization by inducing JC-1 green monomers accumulation (Fig. 5J). In addition, evidenced by the CellROX intensity increase (Fig. 5K), we showed that the TIMM44 blocker provoked ROS production and oxidative injury in HUVECs. Thus, TIMM44 inhibition by MB-10 inhibited endothelial cell functions and angiogenesis.

### Ectopic overexpression of TIMM44 augments endothelial cell angiogenesis in vitro

**Fig. 6:**
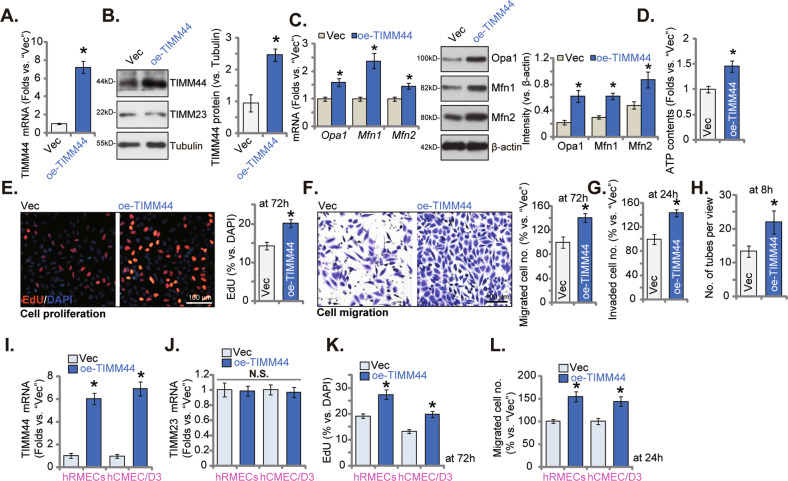
Ectopic overexpression of TIMM44 augments endothelial cell angiogenesis in vitro. HUVECs (**A**–**H**), the human retinal microvascular endothelial cells (hRMECs, **I**–**L**), or hCMEC/D3 brain endothelial cells (**I**–**L**), with the lentiviral TIMM44-expressing construct (oe-TIMM44) or the empty vector (“Vec”), were established, expression of listed mRNAs and proteins was tested (**A**–**C**, **I**, **J**), and ATP contents measured (**D**). HUVECs were further cultivated for indicated time periods, cell proliferation, in vitro cell migration and invasion were examined by the nuclear EdU staining (**E**, **K**), “Transwell” (**F**, **L**) and “Matrigel Transwell” (**G**) assays, respectively, with results quantified; The in vitro capillary tube formation was tested and formed tubes were quantified (**H**). Data were presented as mean ± standard deviation (SD, *n* = 5). *P < 0.05 versus “Vec” cells. “N. S.” stands for non-statistical differences (*P* > 0.05). The experiments were repeated five times with similar results obtained. Scale bar = 100 μm.

To further verify the role of TIMM44 in endothelial cells, the lentivirus encoding the TIMM44-expressing construct was transfected to cultured HUVECs, and stable oe-TIMM44 HUVECs formed after selection. *TIMM44* mRNA (Fig. 6A) and protein (Fig. 6B) levels were indeed robustly elevated in oe-TIMM44 HUVECs, where *TIMM23* mRNA (Fig. 6A) and protein (Fig. 6B) expression was unchanged. *Opa1*, *Mfn1*, and *Mfn2* mRNA and protein levels were elevated in HUVECs with TIMM44 overexpression (Fig. 6C), and ATP contents (Fig. 6D) increased. In HUVECs, oe-TIMM44 promoted cell proliferation and increased EdU-positive nuclei percentage (Fig. 6E). The in vitro cell migration (Fig. 6F) and invasion (Fig. 6G) were accelerated following TIMM44 overexpression in HUVECs. Moreover, the number of formed capillary tubes (per microscope view) was increased in TIMM44-overexpressed HUVECs (Fig. 6H). Thus, TIMM44 overexpression enhanced endothelial cell angiogenesis in vitro. In hRMECs and hCMEC/D3 cells, the application of the lentiviral TIMM44-expressing construct similarly resulted in robust *TIMM44* mRNA overexpression (“oe-TIMM44”) (Fig. 6I), without affecting *TIMM23* mRNA expression (Fig. 6J). Similar to the results in HUVECs, oe-TIMM44 enhanced cell proliferation (Fig. 6K) and in vitro cell migration (Fig. 6L) of the endothelial cells.

### Endothelial knockdown of TIMM44 inhibits retinal angiogenesis in mice

**Fig. 7:**
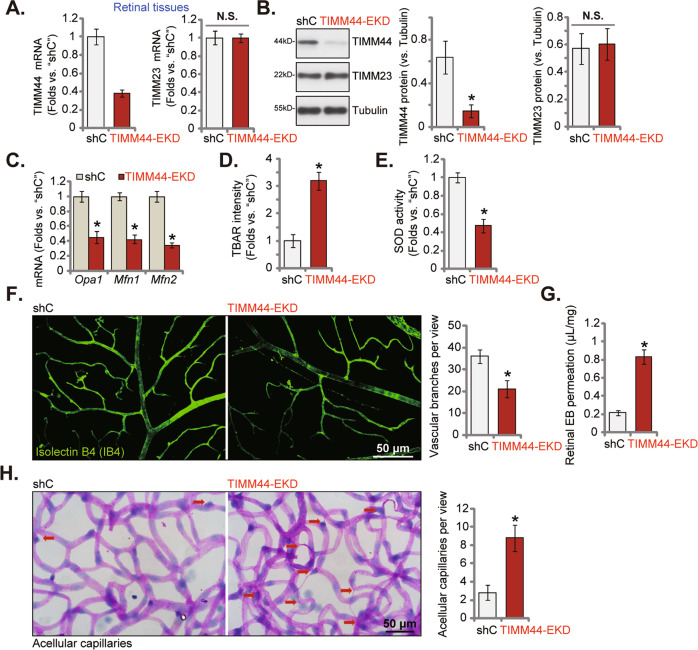
Endothelial knockdown of TIMM44 inhibits retinal angiogenesis in mice. The adult C57BL/6 mice were intravitreously injected with AAV5-TIE1-TIMM44 shRNA (“TIMM44-EKD”, 0.1 μL) or AAV5-packed scramble control shRNA (“shC”, 0.1 μL). After 10 days, the murine retinal tissues were separated and listed mRNAs and proteins in tissue lysates examined (**A**–**C**). The relative TBAR intensity (**D**) and SOD activity (**E**) in the tissue lysates were analyzed as well. IB4 staining was carried out to show retinal vasculature (**F**, scale bar = 50 μm), with the average number of vascular branches per view quantified (**F**). Mice were infused with Evans blue (EB) for 2 h, and Evans blue leakage quantified (**G**). The retinal trypsin digestion was carried out to examine acellular capillaries (red arrows), with their number quantified (**H**, scale bar = 50 μm). The data were presented as mean ± standard deviation (SD, *n* = 5). *P < 0.05. The experiments were repeated five times with similar results obtained.

Retinal vasculature could be directly viewed, offering an accessible window to explore the mechanism of angiogenesis [39]. To explore the potential effect of TIMM44 on angiogenesis in vivo, the adult male C57BL/6 mice were intravitreously injected with AAV5-TIE1-TIMM44 shRNA. It contains sequence of TIE1, the endothelial cell-specific promoter, leading to endothelial knockdown of TIMM44 (“TIMM44-EKD”). The control mice were intravitreously injected with AAV5-TIE1-scramble control shRNA (“shC”). Ten days after the virus injection, retinal tissues were separated and tissue lysates were examined. As shown, *TIMM44* mRNA (Fig. 7A) and protein (Fig. 7B) expression were significantly decreased in TIMM44-EKD retinal tissues, where *TIMM23* mRNA and protein expression was unchanged (Fig. 7A, B). In TIMM44-EKD retinal tissues, *Opa1*, *Mfn1*, and *Mfn2* mRNA levels were significantly decreased (Fig. 7C). Following endothelial knockdown of TIMM44, the TBAR intensity was significantly increased (Fig. 7D) and SOD activity was decreased (Fig. 7E). These results implied that endothelial knockdown of TIMM44 disrupted mitochondrial functions and provoked oxidative injury in retinal tissues.

Significantly, TIMM44-EKD inhibited retinal angiogenesis in vivo. Retinal IB4 staining assay results, Fig. 7F, showed that TIMM44 shRNA AAV injection suppressed retinal angiogenesis in vivo. TIMM44-EKD retinas showed decreased number of vascular branches and branch points (Fig. 7F). Evans blue (EB) results showed that the vascular leakage was significantly increased in TIMM44 shRNA virus-injected retinas (Fig. 7G). The retinal trypsin digestion assay further demonstrated that the number of acellular capillaries was increased in the TIMM44-EKD retinas (Fig. 7H). Thus, endothelial knockdown of TIMM44 inhibited retinal angiogenesis in mice. These results supported the role of TIMM44 on angiogenesis in vivo.

### Endothelial knockdown of TIMM44 results in RGC degeneration in mouse retina

**Fig. 8:**
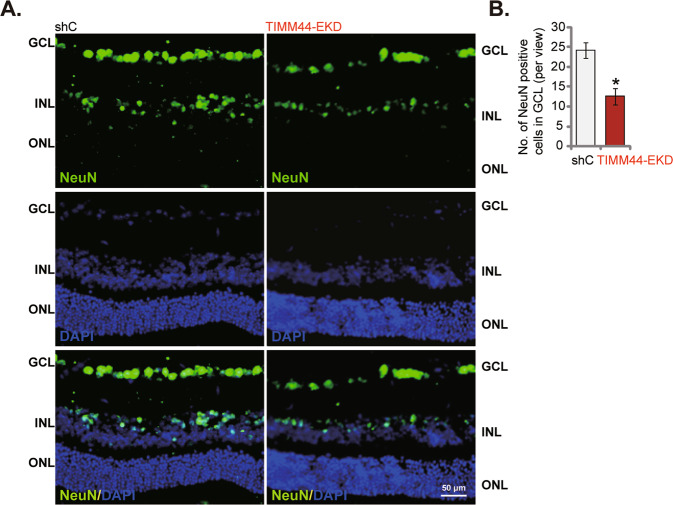
Endothelial knockdown of TIMM44 results in RGC degeneration in mouse retina. The adult C57BL/6 mice were intravitreously injected with AAV5-TIE1-TIMM44 shRNA (“TIMM44-EKD”, 0.1 μL) or AAV5-packed scramble control shRNA (“shC”, 0.1 μL). After 10 days, NeuN/DAPI immunofluorescence staining in mouse vertical retinal slides was shown, and the number of NeuN-positive RGCs in GCL recorded (**A**, **B**, scale bar = 50 μm). The data were presented as mean ± standard deviation (SD, *n* = 5). GCL ganglion cell layer, ONL Outer nuclear layer, INL inner nuclear layer. *P < 0.05. The experiments were repeated three times with similar results obtained.

Angiogenesis inhibition will disrupt supply of oxygen, energy, and nutrients, causing neuronal degeneration in retina [40, 41]. It could be the key pathological mechanism of retinal disorders, including retinopathy of prematurity (ROP), diabetic retinopathy (DR), age-related macular degeneration (AMD), and neovascular glaucoma [42]. We therefore analyzed whether endothelial knockdown of TIMM44 could lead to retinal neuronal degeneration. The adult male C57BL/6 mice were intravitreously injected with AAV5-TIE1-TIMM44 shRNA, causing endothelial knockdown of TIMM44 (“TIMM44-EKD”, see Fig. 7). The vertical retinal immunofluorescence assay results demonstrated that NeuN-positive retinal ganglion cells (RGCs) were decreased following TIMM44-EKD (Fig. 8A, B). These results showed that endothelial knockdown of TIMM44 resulted in RGC degeneration in mouse retina.

### Intravitreous injection of MB-10 inhibits retinal angiogenesis in vivo

**Fig. 9:**
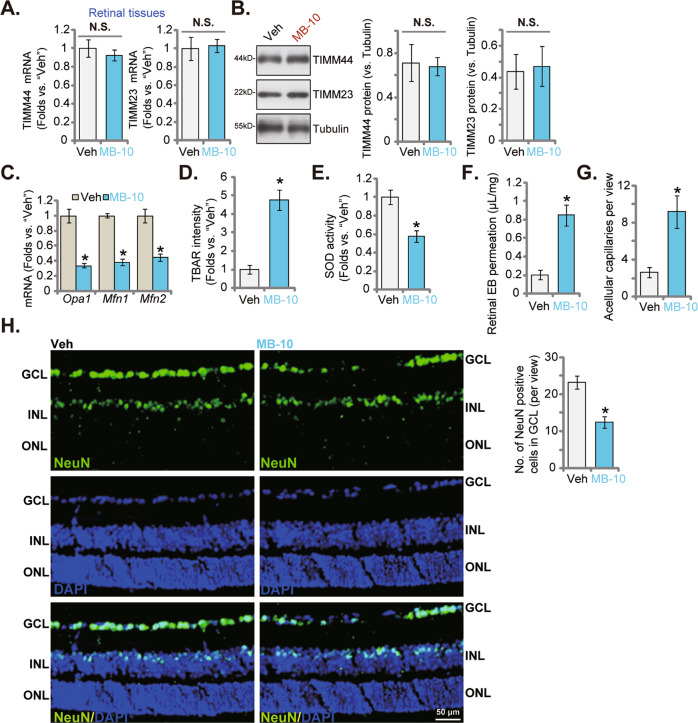
Intravitreous injection of MB-10 inhibits retinal angiogenesis in vivo. The adult C57BL/6 mice were intravitreously injected with MB-10 (0.25 nM) or the vehicle control (“Veh”) for 48 h, the murine retinal tissues were separated and listed mRNAs and proteins in tissue lysates examined (**A**–**C**). The relative TBAR intensity (**D**) and SOD activity (**E**) in the tissue lysates were analyzed as well. Alternatively, 72 h after MB or vehicle administration, mice were infused with Evans blue (EB), with Evans blue leakage quantified (**F**). The retinal trypsin digestion was carried out to examine acellular capillaries (red arrows), with their number quantified (**G**, scale bar = 50 μm). The representative NeuN/DAPI immunofluorescence images of vertical retinal slides were shown, and the number of NeuN-positive RGCs in GCL quantified (**H**, scale bar = 50 μm). The data were presented as mean ± standard deviation (SD, *n* = 5). *P < 0.05. The experiments were repeated five times with similar results obtained.

At last, we explored the potential role of MB-10 on angiogenesis in vivo. The adult male C57BL/6 mice were intravitreously injected with MB-10. After 48 h, retinal tissues were tested. As shown, *TIMM44* mRNA (Fig. 9A) and protein (Fig. 9B) expression was unchanged in MB-10-injected mouse retinal tissues. *TIMM23* mRNA and protein expression was not altered as well (Fig. 9A, B). TIMM44-dependent mRNAs, including *Opa1*, *Mfn1*, and *Mfn2*, were significantly decreased in MB-10-injected retinal tissues (Fig. 9C). Importantly, the TIMM44 blocker caused oxidative injury in retinal tissues, as it increased TBAR intensity (Fig. 9D) but reduced SOD activity (Fig. 9E) in mouse retinal tissues. Retinal angiogenesis was inhibited by intravitreous injection of MB-10 (for 72 h). The quantified EB staining assay results confirmed significantly increased vascular leakage in MB-10-treated retinas (Fig. 9F). Moreover, the relative number of acellular capillaries was increased after treatment with the TIMM44 blocker (Fig. 9G). The retinal immunofluorescence assay results, Fig. 9H, showed that NeuN-positive RGCs were sharply decreased after MB-10 treatment. These results further supported the key role of TIMM44 in angiogenesis in vivo.

## Discussion

A previous study has reported an essential role of TIMM44 in cancer growth [25]. TIMM44 expression is elevated in human glioma, whereas TIMM44 silencing or KO potently suppressed glioma cell growth in vitro and in vivo [25]. Mitochondrial dysfunction was observed in TIMM44-depleted glioma cells [25]. The results of the present study supported that TIMM44 is essential for angiogenesis in vitro and in vivo. Silence of TIMM44 by targeted shRNA or Cas9-sgRNA-induced TIMM44 KO largely inhibited endothelial cell proliferation, migration, and in vitro capillary tube formation. MB-10, a TIMM44 blocker, inhibited proliferation, migration, and in vitro capillary tube formation of endothelial cells as well. Contrarily, ectopic overexpression of TIMM44 augmented pro-angiogenic activity in cultured endothelial cells. In mouse retinas, endothelial knockdown of TIMM44, by intravitreous injection of AAV5-TIE1-TIMM44 shRNA, inhibited retinal angiogenesis in vivo, causing vascular leakage and acellular capillary growth.

The mitochondrial integrity and function are essential for angiogenesis [10, 43–46]. Wang et al. showed that endothelial cell-specific silence of FUN14 domain-containing protein 1 (FUNDC1), a mitochondrial outer-membrane protein, inhibited mitochondria-associated endoplasmic reticulum membrane formation, thereby suppressing VEGFR2 expression, tube formation, spheroid-sprouting, and vessel formation in vitro and in vivo [43]. Ren et al. found that inhibition of pyruvate kinase muscle isoenzyme 2 (PKM2) by C3k in endothelial progenitor cells decreased expression angiogenesis-related genes and inhibited tube formation [45]. Moreover, glycolysis inhibition, mitochondrial membrane potential reduction, ROS production, and oxidative stress were detected in C3k-treated endothelial progenitor cells [45].

We showed that TIMM44 was essential in maintaining mitochondrial functions in endothelial cells. TIMM44 silencing or KO disrupted mitochondrial functions and inhibited mitochondrial protein input, causing mitochondrial complex I activity inhibition and ATP reduction as well as ROS production, mitochondrial depolarization, and apoptosis activation. Moreover, treatment with the TIMM44 blocker MB-10 also induced mitochondrial dysfunction in endothelial cells. Whereas in TIMM44-overexpressed endothelial cells, ATP contents were increased. In mouse retinas, endothelial knockdown of TIMM44 or intravitreous injection of MB-10 induced lipid peroxidation and oxidative injury as well. Thus, TIMM44 depletion/blockage disrupted mitochondrial functions and inhibit angiogenesis in vitro and in vivo.

Studies have discovered that TIMM44-dependent mitochondrial genes, including *Mfn1, Mfn2, and Opa1* [20], are important in regulating angiogenesis [10, 44, 46]. Opa1 expression was increased following angiogenic stimuli, thereby inactivating NFκB cascade to increase angiogenic genes expression, eventually promoting angiogenesis [10]. Chen et al. reported that LncRNA Malat1 promoted angiogenesis by increasing *Mfn1* expression via silencing miR-26b-5p [44]. Contrarily, endothelial cell silencing of Malat1 downregulated *Mfn1* expression, disrupted mitochondrial functions, provoked oxidative stress, and eventually inhibited angiogenesis [44]. Mfn2 is a transmembrane GTPase in the mitochondrial outer membrane. VEGF treatment increased Mfn2 expression in HUVECs. While silence of Mfn2 inhibited VEGF-induced HUVEC migration, indicating a role of Mfn2 in angiogenesis [46].

In the present study, we found that *Opa1*, *Mfn1*, and *Mfn2* expression was significantly decreased in TIMM44-silenced/-KO endothelial cells. Whereas their expression was upregulated in TIMM44-overexpressed endothelial cells. Moreover, TIMM44 blockage by MB-10 led to downregulation of *Opa1*, *Mfn1*, and *Mfn2* in endothelial cells. Endothelial cell-specific knockdown of TIMM44, by intravitreous injected with AAV5-TIE1-TIMM44 shRNA, led to *Opa1*, *Mfn1*, and *Mfn2* mRNA downregulation in retinal tissues. In addition, intravitreous injection of MB-10 downregulated *Opa1*, *Mfn1*, and *Mfn2* expression in retinal tissues as well. Moreover, we found that re-expression of Mfn1, Mfn2, and Opa1 attenuated mitochondrial aberrations and suppression of the angiogenic process in TIMM44-depelted HUVECs. Thus, TIMM44-driven angiogenesis could be due to promoting *Opa1*, *Mfn1*, and *Mfn2* expression. Nonetheless, the concrete mechanism of how TIMM44 regulates angiogenesis needs further investigation.

With disruption of retinal angiogenesis, RGC could be degenerated due to lack of oxygen, nutrition, and energy supply and by the inflammatory environment [47–49]. This could be a key pathological mechanism of retinal vascular diseases, including DR, AMD, and neo-vascular glaucoma [47–49]. We have previously shown that endothelial knockdown of phosphoenolpyruvate carboxykinase 1 (PCK1) suppressed retinal angiogenesis in mouse, causing significant RGC degeneration [29]. In the present study, we found that endothelial knockdown of TIMM44 or intravitreous injection of MB-10 resulted in robust RGC degeneration in mouse retinas. Thus, TIMM44 depletion/blockage inhibited retinal angiogenesis, leading to RGC degeneration in mouse retinas.

## Conclusion

Recent studies have supported that mitochondria are essential in maintaining endothelial cell functions [11, 12, 50]. However, mitochondrial proteins that are critical for angiogenesis remain largely unknown [11, 12, 50]. We conclude that the mitochondrial protein TIMM44 is important for angiogenesis in vitro and in vivo. Future studies will be needed to explore other mitochondrial proteins, in this or other cascades, that are vital for the regulation of angiogenesis.

## Supplementary information

Figure S1

Figure S2

aj-checklist form

## Data Availability

All data generated during this study are included in this published article. Data will be made available upon request.
