# Supplementary material for: The mitochondrial protein TIMM44 is required for angiogenesis in vitro and in vivo
Source: Cell Death Dis. 2023 May 5;14(5):307. doi: 10.1038/s41419-023-05826-9 (PMC10163060; doi:10.1038/s41419-023-05826-9)

Figure S1: The uncropped blotting images.

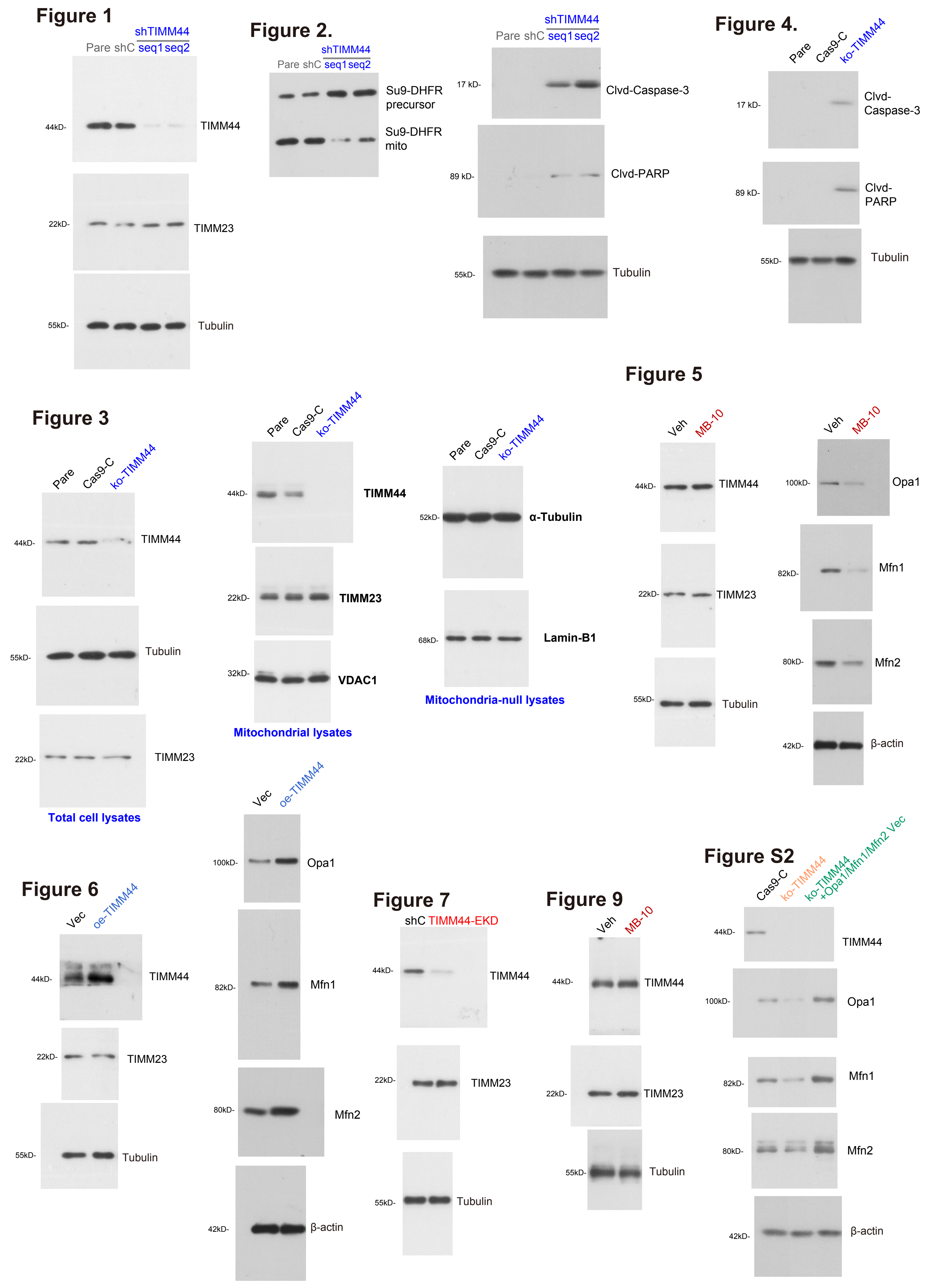

Supplement: Supplementary file 1 — Figure S1 [file 41419_2023_5826_MOESM1_ESM.pdf]
