# Supplementary material for: The mitochondrial protein TIMM44 is required for angiogenesis in vitro and in vivo
Source: Cell Death Dis. 2023 May 5;14(5):307. doi: 10.1038/s41419-023-05826-9 (PMC10163060; doi:10.1038/s41419-023-05826-9)
Supplement: Supplementary file 2 — Figure S2 [file 41419_2023_5826_MOESM2_ESM.pdf]

Figure S2

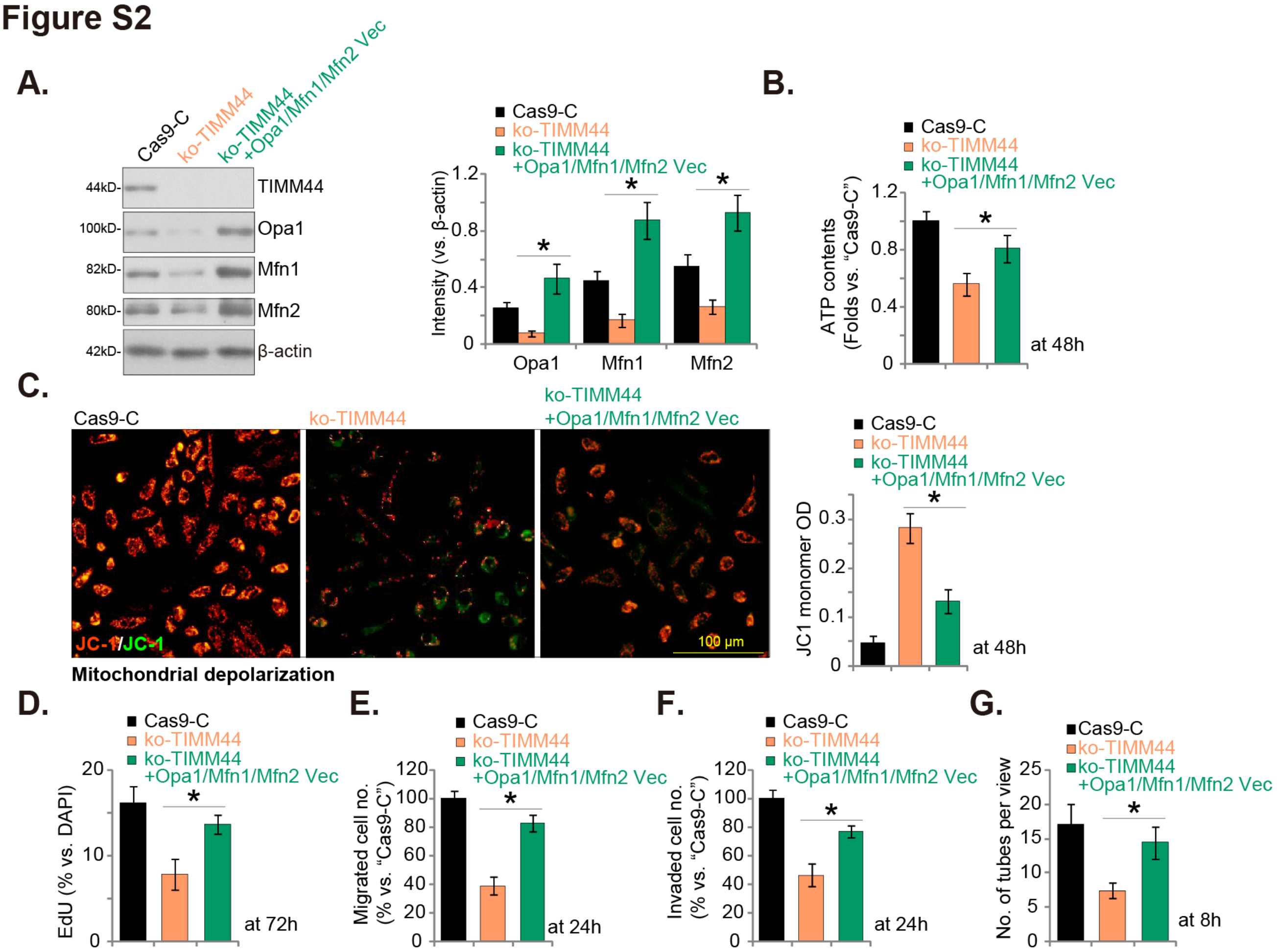

Figure S2. HUVECs, expressing the CRISPR/Cas9 empty vector ("Cas9-C"), the Cas9 construct plus the CRISPR/Cas9-TIMM44-KO construct ("ko-TIMM44") or ko-TIMM44 plus the Opa1-expressing lentiviral construct, the Mfn1-expressing lentiviral construct and the Mfn2-expressing lentiviral construct ("ko-TIMM44+Opa1/Mfn1/Mfn2 Vec") were cultivated and expression of listed proteins was shown (**A**); ATP contents (**B**), the mitochondrial membrane potential reduction (JC-1 staining, **C**), cell proliferation (EdU-positive nuclear ratio, **D**), *in vitro* cell migration (**E**) and invasion (**F**) and formed capillary tubes (per view, **G**) were examined, with results quantified. Data were presented as mean ± standard deviation (SD, n = 5). * ***P*** < 0.05. The experiments were repeated five times with similar results obtained. Scale bar = 100 μm.
